# Supplementary material for: Patterns and risk factors of opioid-suspected EMS overdose in Houston metropolitan area, 2015-2019: A Bayesian spatiotemporal analysis
Source: PLoS One. 2021 Mar 11;16(3):e0247050. doi: 10.1371/journal.pone.0247050 (PMC7951926; doi:10.1371/journal.pone.0247050)

S1 Fig: Map of the study region which includes 84 zip code areas within Hwy 610 belt in Houston, Texas. Total counts here the opioid-suspected overdose from EMS calls between 2015 and 2019.


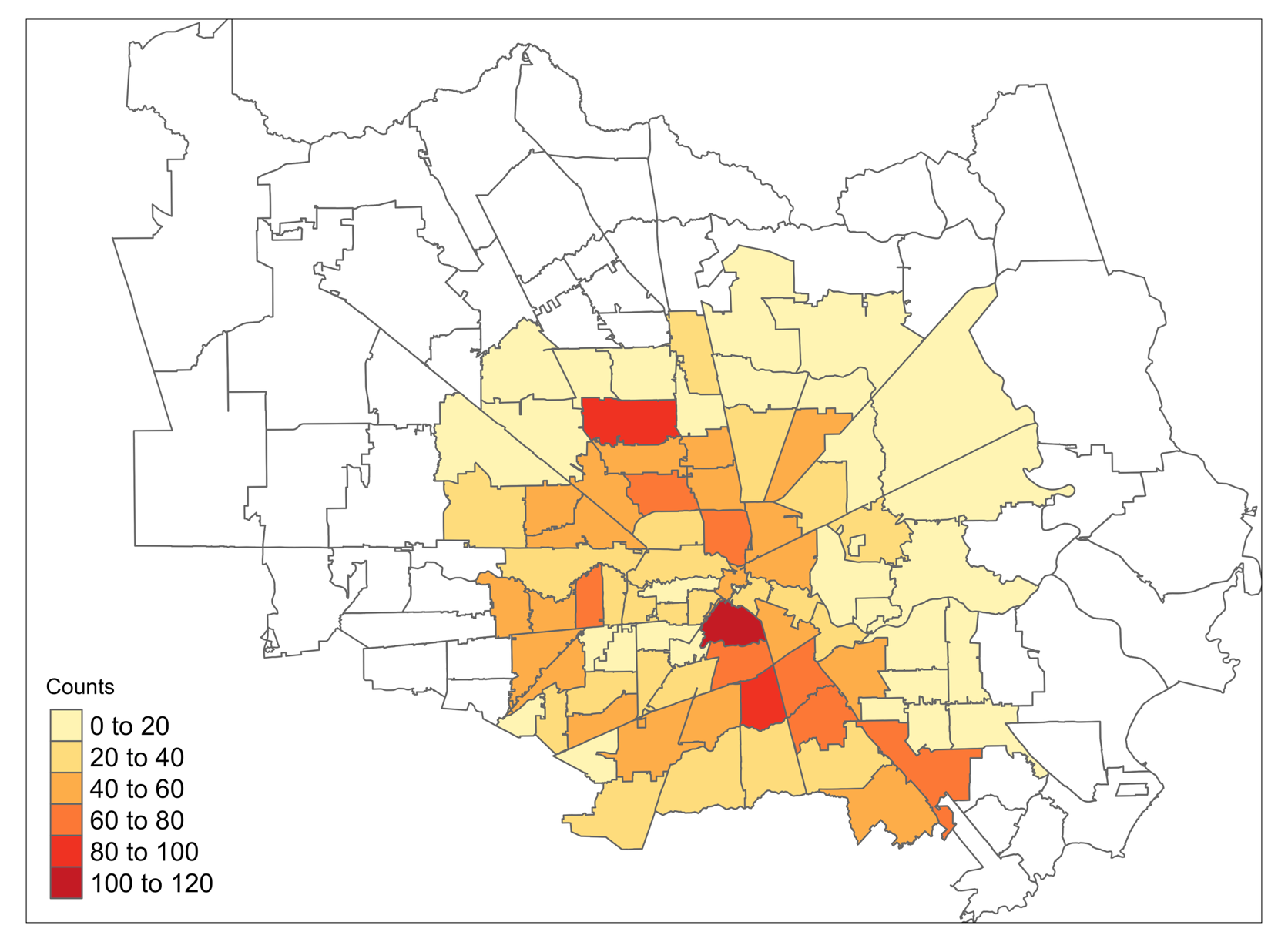

Supplement: S1 Fig — Counts are the total opioid-suspected overdose from EMS calls between 2015 and 2019. (DOCX) [file pone.0247050.s001.docx]
