# Supplementary material for: Patterns and risk factors of opioid-suspected EMS overdose in Houston metropolitan area, 2015-2019: A Bayesian spatiotemporal analysis
Source: PLoS One. 2021 Mar 11;16(3):e0247050. doi: 10.1371/journal.pone.0247050 (PMC7951926; doi:10.1371/journal.pone.0247050)
Supplement: S2 Fig — (DOCX) [file pone.0247050.s002.docx]

S2 Fig. Correlation of the zip code SES variables within study region.
