# Supplementary material for: Patterns and risk factors of opioid-suspected EMS overdose in Houston metropolitan area, 2015-2019: A Bayesian spatiotemporal analysis
Source: PLoS One. 2021 Mar 11;16(3):e0247050. doi: 10.1371/journal.pone.0247050 (PMC7951926; doi:10.1371/journal.pone.0247050)
Supplement: Attachment — Submitted filename: Response to Reviewers.docx [file pone.0247050.s004.docx]

Dear Dr. Zaller,

Thank you for providing us the opportunity to submit a revised version of our manuscript titled “Patterns and risk factors of opioid-suspected EMS overdose in Houston Metropolitan area, 2015-2019: A Bayesian Spatiotemporal Analysis” to *Plos One*. We are grateful to the reviewers for their insightful comments and their valuable suggested edits and revisions. In addressing the reviewer comments, we believe the manuscript is much improved.

Below is a point-by-point response to the reviewers’ comments and recommendations. We hope that our revision has addressed the reviewers’ concerns and comments. Thank you again for reviewing our submission.

**Comments from the Editor**

**Comment 1**: Please ensure that your manuscript meets PLOS ONE's style requirements, including those for file naming.

**Response:** We have ensured that our revised submission meets PLOS ONE’s style requirements.

**Comment 2**: Data upon request is only allowed if there are legal or ethical restrictions on sharing data publicly.

a) If there are ethical or legal restrictions on sharing a de-identified data set, please explain them in detail (e.g. data contain potentially identifying or sensitive patient information) and who has imposed them (e.g. an ethics committee). Please also provide contact information for data access committee, ethics committee, or other institutional body to which data requests may be sent.
b) If no restrictions, please upload the minimal anonymized data set necessary to replicate findings.

**Response:** The data used in this analysis contains private health information that is considered confidential. The de-identified data contains information on location and medical diagnosis and therefore cannot be made public due to the risk of breach of confidentiality.

**Comment 3:** PLOS requires an ORCID iD for the corresponding author in Editorial Manager on papers submitted after December 6th, 2016. Please ensure that you have an ORCID iD and that it is validated in Editorial Manager.

**Response:** The ORCID ID for the corresponding author (Dr. Langabeer) has been validated and is linked to his Plos One username. It is 0000-0002-2304-4853.

**Comment 4:** Please ensure that you refer to Figure 3 in your text to link the reader to the figure.

**Response:** We have included in the revised submission the following text directing the reader to Figure 3, as the first sentence in the second paragraph under Results section:

“The observed SIRs of opioid-suspected overdose EMS incidents are shown in Figure 3(A), where we clearly observed the spatial patterns.” In addition, we have also corrected the sentence when referencing the model-based estimates in “Model-based zip code level RR from the BYM model was presented in Figure 3(B)” in the same paragraph.

**Comment 5:** Figures 1 and 3 may contain images (map/satellite) which may be copyrighted. All PLOS content is published under the Creative Commons Attribution License (CC BY 4.0) which means the manuscript, images and supporting info files will be freely available online and any third party is permitted to access, download, coy, distribute and use these materials in any way, even commercially, with proper attribution. For these reasons, we cannot publish previously copyrighted maps or satellite images created using proprietary data, such as Google software (Google Maps, Street View, Earth).

a. We require you either present a written permission from the copyright holder to publish these figures under the CC BY 4.0 license or remove them from your submission. Please upload the permission form.

**Response:** All data and images were created using R, an open-source software environment that is available at no cost under the terms of the Free Software’s Foundation’s GNU General Public License. As such, the analysis, including images are the property of the user.

Citation: Lovelace, R.; Nowosad, J., & Muenchow, J. (2020). Geocomputation with R. Accessed on October 26, 2020 from: <https://www.r-project.org/about.html>

Free Software Foundation, Inc. (1991). GNU General Public License. Accessed on October 26, 2020 from: <https://www.r-project.org/COPYING>.

**Comments from Reviewer #1:**

**Comment 1**: Make it clearer how the spatiotemporal models could help with local efforts to combat the opioid epidemic over more real-time displays of the actual EMS data.

**Response:** There are several reasons that model based approaches (including the Bayesian spatiotemporal models we adopted in this analysis) are better than just using the displays (for example maps) of the actual EMS data. First, for less populated areas, the observed data, or estimates only using the observed data, may not capture the true underlying incidence risk. For example, if there are zero EMS calls in a given ZIP code from the study region, the maps displaying the observed EMS data will show the risk of having EMS calls is zero. However, we would NOT conclude that the incidence risk of having EMS would be zero, or forecast the future risks would be zero. Second, the observed EMS data often present a lot of noise, and model-based approaches allow us to tease out the noise and reveal the true underlying pattern, and to identify “hot spots” of disease incidence and forecast risks in order to focus intervention and preventative measures. Third, the model-based approaches allow us to investigate the potential contributing factors to the “hotspots”, that can inform policy and public health intervention. For example, in our analysis, we identified the “hotspots” are associated with areas with a higher percentage of home renters (rather than owners), therefore, for future intervention, we may specifically focus on dense areas with large proportions of apartment complexes. Finally, the model-based approaches can provide prediction of future hotspots, which can aid the resource allocation and distribution of state and federal funds to combat the opioid crisis.

In the revised submission, we have further clarified the benefits of using model-based approach over the observed estimates in the Introduction section.

**Comment 2**: Abstract methods: include some general demographic info about the cohort along with explicit statement this is a cross sectional analysis.

**Response**: We have made the change in the manuscript to reflect the cross-sectional study design. Unfortunately, the EMS data we utilized do not report details on the demographic information. To clarify with the study design, the following text was added to the abstract methods section and the main text:

Using a cross sectional design and Bayesian spatiotemporal models, we identified zip code areas with excessive opioid-suspected incidents, and assessed how the incidence risks were associated with zip code level socioeconomic characteristics. The study population was approximately 24% white, and 48% Hispanic. Other racial characteristics and gender were not included.

**Comment 3**: Conclusions: include a clinically relevant insight from the results section. Overall, what do the results tell us about how to focus our community overdose strategies?

**Response:** This was addressed in revisions to parts of the discussion section that are detailed in this document. We added implications for response and prevention mitigation efforts, forecasting of problem areas, focusing on specific population demographic trends (youth, elder), funding allocation, staffing considerations.

**Comment 4:** Introduction—a sentence about the availability of real-time EMS data could be helpful in knowing how easy it would be for localities to adopt the use of that data. Is most EMS data across the country lagging in time?

**Response:** The following text in the second paragraph of the Introduction was revised to include limitations of real-time EMS data:

“Monitoring of real-time drug overdose patterns is an essential tool in combatting the epidemic; yet, federal and state surveillance systems have considerable limitations. National drug surveillance systems which rely on a top-down approach, are slow to report patterns, and can even lag behind changing overdose trends (6). Additionally, state and local level EMS data collection systems are not standardized. EMS agencies differ drastically in resources and local EMS agencies with limited resources may be reluctant to invest in more sophisticated data collection software. Due to the variability in collection practices, researchers often rely on field notes and data captured by dispatch systems.”

**Comment 5:** Cross-sectional study design?

**Response:** Yes, this is a cross-sectional study design and we have clarified it in the revision.

**Comment 6:** This sentence isn’t clear to me: “Data from 2015 to 2018 were included; however, data from 2019 was not published and so we used data from 2018 for 2019.”

**Response:** The data referenced here are ACS data and at the time of this analysis was conducted, ACS data (e.g., population size, percentage of unemployed) were only available for year 2015, 2016, 2017 and 2018 but not for 2019, and so we assumed that the ACS variables in 2019 were the same as those from 2018. We have edited the sentence to make it clear.

**Comment 7:** It might help someone like me who is not fluent in Bayesian models to include a citation on why these three model selection criteria were used.

**Response:** These model selection criteria were among the most commonly used ones in Bayesian analysis, and we now included citation to provide additional details.

**Comment 8:** I think there is an extra ‘9’ in this sentence: “We observed a slight decrease in 2017 9 (n=473) compared to 2016 (n=487), 123 with a significant increase in calls in 2018 (n=555) and again in 2019.

**Response:** Thank you for pointing out the typo. This has been corrected as indicated in the text below:

“EMS calls for opioid-suspected overdose incidence increased from 449 in 2015 to 666 in 2019 (Figure 2). We observed a slight decrease in 2017 (n=473) compared to 2016 (n=487), with a significant increase in calls in 2018 (n=555) and again in 2019.”

**Comment 9:** Are there other zip code level data sources other than ACS that could be incorporated into the analysis (e.g. distressed community index)?

**Response:** Technically, we can include other community index in the analysis, as long as they are also available at the zip code level. We chose to only consider the individual ACS variables because other community index are often constructed using the ACS variables, and hence are highly correlated with the ACS variables.

**Comment 10:** Do we have any citations on how often an overdose occurs where EMS is not called?

**Response:** Unfortunately, it is difficult to track how often an overdose occurs when EMS is not called, and we do not have this information in our analysis. However, several studies have reported large percentages of overdose survivors do not get treated by EMS (Knowlton, 2013) and EMS call surveillance can provide the data necessary to fill in the data gaps in identifying overdose trends. We have included the additional discussion in the revision.

Citation: Knowlton A, Weir BW, Hazzard F, et al. EMS runs for suspected opioid overdose: implications for surveillance and prevention. Prehosp Emerg Care. 2013 Jul-Sep 2013;17(3):317-329.

**Comment 11:** In the discussion of the programs that use EMS data (e.g., DOPE), it would be good to expound upon the paragraph talking about why geospatial modeling could provide a more comprehensive response to the epidemic. How could this be more helpful? What specific aspects?

Response: We have included the following text in the revision:

“Geospatial analysis of the most current data on suspected opioid-overdose calls can inform community programs on trending hot spots but can also help target specific populations that are experiencing increased overdose events by including certain demographic characteristics in the analysis. Interventions that target special populations, such as youth, females of childbearing age, and elder populations can use geospatial analysis to narrow their target areas.”

**Comment 12:** I would include more of a discussion of the months and time of day that overdoses occur. This could help with EMS staff allocation.

**Response:** The time and months of the EMS on overdoses was described in Figure 2. Temporal characteristics of opioid-suspected EMS calls. In the revision, we have added the following text:

“Additionally, temporal trends can be tracked and help inform staffing practices of EMS providers. Our analysis revealed more overdoses occur in the summer months, between May and September and between 7pm and 11pm. This information, combined with geospatial trends can assist municipalities with hiring and staffing practices, ensuring enough adequately trained personnel are on duty during those high-risk times.”

**Comment 13:** It is still unclear to me how this model would be more beneficial than real-time EMS data? What does this offer over systems to display real-time data on overdoses?

**Response:** We hope that our response to Comment 1 has now clarified the benefits of model-based approaches. But, to be clear, communities need proactive strategies for detecting changes in where people are dying and overdosing for public health program planning. Strategies that communities can deploy to provide health promotion and education to geographic clusters that are predicted as high-risk, can help to potentially thwart the opioid epidemic. We added the following verbiage to the Discussion section in the revised manuscript:

“Spatiotemporal modeling provides advantages over monitoring real-time EMS call data. First, it helps visualize a complex and dynamic problem that fluctuates across time and space. Geospatial analysis can actualize changes across time spans, from days to years and can identify patterns to assist with causation research. Spatiotemporal models can help inform policy makers with budget allocation and staffing concerns. Applying a Bayesian approach can examine existing trends and apply them to forecast patterns, which is useful for prevention efforts. Altogether, Bayesian spatiotemporal modeling can help EMS providers, researchers, and lawmakers refine programs and operate in a constantly changing environment.”

**Comment 14:** Expound upon this sentence from the Results in the Discussion: “We also observed substantial changes in the spatial variation over time, which provided strong evidence to include spatiotemporal interaction in the Bayesian spatiotemporal models.” How is this beneficial?

**Response:** If the spatial patterns does NOT change over time, we will adopt a spatiotemporal model and essentially assume the same spatial pattern for each year. This is to say that, hotspots will always be the same areas, no matter which year we are looking at. Since the spatial variation clearly changes over time, we cannot assume the same spatial pattern for every year and hotspots may change over time. Therefore, we need to include a spatiotemporal interaction term in the spatiotemporal model. To expand on this point we have added the following sentence:

“Without the interaction term, we essentially assume the same spatial pattern and hotspots every year. With the interaction term in the model, we capture the changing spatial pattern over time and allow different hotspots every year”.

**Comment 15:** I would consider adding a limitation that this analysis does not likely represent rural areas.

**Response:** We have included the following text in the revised submission:

“ . . nor do our results apply to rural areas which may have different demographics and risk factors.”

**Comment 16:** Do you have any estimates of how much the convenience sample issue may cause errors?

**Response:** The data we used is not a sample but rather from an observational study. It is possible the an overdose occurs where EMS is not called, and hence not captured in our dataset. We have addressed this limitation in the revised manuscript (also see our response to Comment 10 above and comment 17 below).

**Comment 17:** Do you have an estimate of how often overdoes occur but are not called into 911? If so, that would be good to add with the last limitation.

**Response:** We hope that our response to Comment 8 above has addressed your question.

**Comments from Reviewer #2:**

This spatial ecological panel analysis uses Emergency Medical Service data for opioid overdoses in Houston, aggregated within 84 ZIP codes over 5 years (n = 420 ZIP code-years). The authors use Bayesian conditional autoregressive models to assess the geographic distribution of opioid overdoses in relation to demographic characteristics, as measured using American Community Survey data. The statistical analyses are very well constructed and presented, and I commend the authors for their meticulous work and clear explanations. Nevertheless, I think the basic framing of the paper needs some attention, and there are some other issues that detract from an otherwise elegant statistical analysis.

**Major issues:**

**Comment 1:** The main problem is that the aim of the paper (to demonstrate use of Bayesian spatial methods as applied to this particular outcome) is not a terribly compelling scientific justification for the work. These models have been around for some time and they’ve been applied to many different outcomes. I don’t think there would be any doubt that they can be used for opioid overdoses, of that there will be geographical variation in overdoes. Absent a convincing rationale, the analysis becomes demographic predictors. The following steps are suggested:

a) Propose and test a clear theoretical mechanism that would cause opioid overdose to concentrate in areas with specific social conditions.
b) Justify theoretically the spatial and temporal scale of the analyses? (why zip codes and why years)?
c) Consider adding other areal characteristics that might be theoretically related to outcome, beyond basic demographics.

**Response:** Thank you for your comments and suggestions. We did not set out to justify the utility of Bayesian spatiotemporal models in the context of opioid-related EMS overdose. But rather, we use this modeling tool to better understand the geographic and spatial patterns that are occurring in a very large city experiencing high rates of overdoses. We used these analyses to identify the high-risk geographical areas for future intervention design and policy making, which is the main component of our manuscript. The mechanism of what “causes” opioid overdose was complicated and with an observational study, we will not be able to conclude the causal relationship of the opioid overdose and the zip code characteristics (if any). The spatial and temporal patterns from our analysis, and a common goal of using spatiotemporal models would be to assist generating hypothesis. We included area level social conditions (e.g., ACS variables) as exploratory analysis to understand the factors associated with the spatial pattern, but testing the causal mechanism is beyond the scope of this work. Though the models themselves are not new, we do believe, however, that our analysis is quite novel, as there has been no prior studies that utilized similar methods to address the opioid epidemic, particularly to the population we are studying.

The reasons that we chose zip code and years as our spatial and temporal units are partially due to data availability, and partially due to the interest of the end users of our analysis. We collaborated with and obtained the data from the Houston fire department, and the chosen spatial and temporal scale was preferred by them for future resource allocation and intervention planning.

The ACS variables we included are not just basic demographics but also covered other social domains. These variables were carefully chosen based on our scientific interest and findings from previously studies on this topic. In addition, these variables are also directly related to the

strategies that communities can deploy to provide health promotion and education to geographic clusters that are at high risk of opioid-related overdose, potentially assisting in thwarting the opioid epidemic.

**Comment 2:** The demographic characteristics are likely to be correlated with one another. I suggest presenting a correlation matrix or using some data reduction approach to combine these variables in a meaningful way.

**Response:** We now included a correlation matrix of the ACS variables in the supplementary materials, and it is not surprising that some ACS variables are correlated. We chose to investigate the individual ACS variables instead of using some data reduction approach for three reasons: 1) despite some correlation, we are dealing with a reasonable number of covariates that, with our sample size and modeling approach, allows us to investigate association; 2) the association with individual ACS variables (e.g., percentage of renters or blue collars) are of our research interest, as they better inform us with the future design of intervention and outreach programs; 3) what would be a “meaningful way” to combine the ACS variables is debatable and depends on the research question. In our case, we feel the individual ACS variables are actually more meaningful in our research to understand the variation in opioid-related EMS calls. Therefore, we decided to keep our current approach using the ACS variables.

**Comment 3:** The emphasis in the Introduction section on EMS data as a rapid and comprehensive data source seems to set up a surveillance study. A few lines to justify the data source is plenty for an analytic study that addressed associations between an exposure and an outcome.

**Response:** It is unclear to us why this is an exposure and outcome analysis, and the need to justify EMS data in this context. It is common practice to use data from EMS, fire departments, and police departments to examine patterns across space and time, identify areas with high EMS calls and investigate what area-level characteristics may be associated with EMS incidence.

**Minor issues:**

**Comment 4:** Line 53 - which university?

**Response:** We have corrected the information to “University of Texas Health Science Center at Houston”.

**Comment 5:** The methods section should note early that the temporal partition is by year.

**Response:** The spatial and temporal unit used in this analysis is clarified in the Methods section describing the Bayesian spatiotemporal models.

**Comment 6:** Line 62 - “data from 2015 to 2018 were included; however, data from 2019 was not published and so we used data from 2018 for 2019” sentence not clear.

**Response:** The data referenced here are ACS data and at the time of this analysis was conducted, ACS data (e.g., population size, percentage of unemployed) were only available for year 2015, 2016, 2017 and 2018 but not for 2019, and so we assumed that the ACS variables in 2019 were the same as those from 2018. We have clarified the sentence as “ACS data were only available for years between 2015 and 2018, and so we used the data from 2018 for 2019.”

**Comment 7:** It’s important to note whether the ACS data are 1-year estimates or 5-year estimates.

**Response:** For this study, we used the American Community Survey 5-Year estimates from 2015 to 2018. We have clarified the ACS data source in this revision.

**Comment 8:** Line 89 – why is the smoothed rate considered more accurate?

**Response:** The smoothed rate is more accurate than the observed rate for the following reasons. First, for less populated areas, the observed data, or estimates only using the observed data, may not capture the true underlying incidence risk. For example, if there were zero EMS calls in a given ZIP code from the study region, the observed rate would be zero. However, the underlying rate could be very small but certainly NOT zero. Second, the observed rates often present a lot of noise, and smoothed rate would be able to tease out the noise and reveal the true underlying pattern, by borrowing information across time and over space. We have articulated this point in the revised Introduction section.

**Comment 9:** Equation 1: Is there a reason you didn’t include a time trend? Or perhaps there is a reason you wanted that variation to be captured in the error term?

**Response:** You are correct that the time trend is captured in the error/interaction term. Since we modeled the annual trends with 5 years, we didn’t want to make any strong assumption of the temporal trend (e.g., linear or quadratic) and hence we decided to use a “catch-it-all” term in the interaction term.

**Comment 10:** Line 118 - should say that INLA approximates the MCMC

**Response:** We have made this note in the revision, and added the relevant reference.

**Comment 10:** Figure 3 – Y-axis should be on the log scale for ratios

**Response:** It is standard practice in spatiotemporal analysis to present relative risk (RR) on the exponentiated scale. As we stated in the manuscript that the reason we chose to use SIR and RR is their easy interpretation: SIR or RR > 1 means more observed counts than the expected, and hence “hot spots.” The scale we adopted in Figure 3 actually reflects this interpretation so we think it is best to keep the Y-axis in Figure 3 as is.
